# The Prince’s Escape

**Published:** 1883-09

**Authors:** 


### THE PRINCE'S ESCAPE.

AMONG the prominent men of the Nihilists is Prince Krapotkine, a refugee, whose revolutionary acts have exiled him from Russia. He belongs to the highest Russian aristocracy, and is the author of several works highly appreciated by scientific men.

While he was chamberlain to the late Empress of Russia, he became an Internationalist and advocated the anarachical ideas of the extreme Socialists. A series of secret lectures in socialism and revolution, which he delivered to workmen, led to his arrest. The Russian Court was amazed that such a high personage should be connected with the revolutionary party.

The Prince was imprisoned in the fortress of St. Peter and St. Paul, whence escape seemed impossible. But he did escape, after three years' confinement. The plan of the escape, a masterpiece of accurate calculation and resolution, and drawn up by himself, he once described to a group of friends. He said :

The prison having undermined my health, I was transferred to the St. Nicholas Hospital. In a few months it was re-established, but I did everything to hide the fact.

I walked with the step of a dying man and spoke with a low voice. My object was to prolong my stay in the hospital, where the surveillance was less strict than in the fortress, as I learned that an attempt was being organized to effect my escape.

The doctor ordered me daily exercise. At one o'clock I was taken into the large court-yard of the hospital. A sentinel, musket in hand, was always by my side.

The gate was then open, for the supplies of wood for the winter were being taken in. No sentinel was placed at the gate.

I walked up and down at the bottom of the court-yard, exactly opposite the gate. The sentinel was always near, between me and the gate. As my slow walking wearied him, he followed a line parallel to mine, but five paces nearer the gate. He thus made his walk ten paces longer than mine, or at each extremity of his line he was always at the same distance from the gate as I was at the extremity of my line.

I thought that if we both began to run, the soldier, by a natural instinct, would try to seize me as quickly as possible, and would therefore rush upon me, instead of running directly to the gate to cut off my retreat.

He would thus describe two sides of the triangle, of which I should describe the third. I might hope, therefore, to reach the gate before the sentinel, running at the same speed.

I arranged through friends that a vehicle should await me, on a given day, at the gate, and a friend should post himself there to assist me in getting in quickly.

The invalid's dressing-gown, which I wore in the hospital, was so large and long, that in walking I was obliged to carry my train upon my arm. To run in such a garb was impossible.

It must be thrown off, and this must be done with the rapidity of lightning. I practiced this in my cell.

The selection of the moment was the greatest difficulty. This depended upon the condition of the narrow, winding street through which we had to pass. A string of wood carts, passing soldiers, or a mounted cossack might upset the attempt.

Sentinels were placed to watch the streets. A friend hired a room on the third story directly opposite the hospital. From the window could be seen the sentinels and the court-yard. The signal that all was clear was to be given by the playing of a violin, and the music would cease when anything unfavorable occurred.

I went to take my exercise. No sooner had I entered the court-yard than I heard the violin. The music lasted for five minutes, and then ceased. Two minutes afterward some carts with wood entered the court-yard. The violin began again.

I looked at the sentinel ; he was walking along his line, some five paces distant, between me and the gate. "Now or never," I said to myself. I seized my dressing-gown—the violin ceased. I felt as if I should drop. A moment after the music began again ; a patrol had passed.

I threw off my dressing-gown and was off like an arrow. The sentinel, with a howl, rushed at me instead of running straight to the gate.

On reaching the gate I saw a vehicle. A friend, dressed as an officer, thrust me in the carriage, which went off like a flash of lightning.

At the hospital there was an uproar. The officer of the guard tore his hair, exclaiming, "I am ruined ! Run after him ! "

My friend who had played the violin descended into the street and asked the officer what had happened. The frenzied officer lost time in replying to him.

An old woman said, "They will go a roundabout way, and then make straight for the Nevski. Take out the horses from these omnibuses at the gate and cut off their escape."

This was exactly the course we were taking, but the terrible advice of the old crone was not followed.

---

Schools, as a rule, are very defectively ventilated. Ordinarily flat ceilinged rooms are totally unfit for public schools. The space should be open up to the roof ridge, and this should be covered.

